# Author Correction: Evolutionary roots of the risk of hip fracture in humans

**DOI:** 10.1038/s42003-025-08498-7

**Published:** 2025-07-18

**Authors:** Hadas Leah Levine, Nir Shvalb, Ariel Pokhojaev, Samuel Francis, Ruth Pelleg-Kallevag, Victoria Roul, Jean-Jacques Hublin, Frank Rühli, Hila May

**Affiliations:** 1 Department of Anatomy and Anthropology, Sackler Faculty of Medicine, Tel Aviv University, Tel Aviv, 6997801 Israel; 2 The Shmunis Family Anthropology Institute, the Dan David Center for Human Evolution and Biohistory Research, Sackler Faculty of Medicine, Tel Aviv University, Tel Aviv, 6997801 Israel; 3 Mechanical Engineering Department, Ariel University, Ariel, 40700 Israel; 4 Department of Oral Biology, The Maurice and Gabriela Goldschleger School of Dental Medicine, Tel Aviv University, Tel Aviv, 6997801 Israel; 5 Zefat Academic College, Zefat, Israel; 6 Chaire de Paléoanthropologie, CIRB (UMR 7241 – U1050), Collège de France, Paris, 75231 France; 7 Max-Planck Institute for Evolutionary Anthropology, Leipzig, 04103 Germany; 8 Institute of Evolutionary Medicine, University of Zurich, Zurich, CH-8057 Switzerland

**Keywords:** Biological anthropology, Risk factors

Correction to: *Communications Biology* 10.1038/s42003-023-04633-4, published online 17 March 2023

In this article the author name Hadas Leah Levine was incorrectly written as Hadas Leah Avni. The original article has been corrected.

