# Supplementary material for: Evolutionary roots of the risk of hip fracture in humans
Source: Commun Biol. 2023 Mar 17;6:283. doi: 10.1038/s42003-023-04633-4 (PMC10023703; doi:10.1038/s42003-023-04633-4)
Supplement: Supplementary file 2 — Supplementary Information [file 42003_2023_4633_MOESM2_ESM.pdf]

Supplementary information

Evolutionary roots of the risk of hip fracture in humans

Hadas Leah Avni[1,2], Nir Shvalb[3], Ariel Pokhojaev[1,2,4], Samuel Francis[1,2], Ruth Pelleg-Kallevag[1,2,5], Victoria Roul[1,2], Jean-Jacques Hublin[6, 7], Frank Rühli[8], and Hila May[1,2]*

[1]Department of Anatomy and Anthropology, Sackler Faculty of Medicine, Tel Aviv University, Tel Aviv 6997801, Israel

[2]The Shmunis Family Anthropology Institute, the Dan David Center for Human Evolution and Bio-history Research, Sackler Faculty of Medicine, Tel Aviv University, Tel Aviv 6997801, Israel

[3]Mechanical Engineering Department, Ariel University, Ariel 40700, Israel.

[4]Department of Oral Biology, The Maurice and Gabriela Goldschleger School of Dental Medicine, Tel Aviv University, Tel Aviv 6997801, Israel

[5]Zefat Academic College, Zefat, Israel.

[6]Chaire de Paléoanthropologie, Collège de France, Paris 75231, France.

[7]Max-Planck Institute for Evolutionary Anthropology, 04103 Leipzig, Germany.

[8]Institute of Evolutionary Medicine, University of Zurich, Zurich CH-8057, Switzerland.

* Corresponding author: Dr. Hila May, Department of Anatomy and Anthropology, Sackler Faculty of Medicine, Tel Aviv University; e-mail: mayhila@tauex.tau.ac.il

1. **Supplementary Tables**
2. **Supplementary Figures**

1. Supplementary Tables

Table S1| The Levantine ancient sample included in the study

Period	Subsistence strategy	Archeological site	Estimated age at death/life expectancy for adults	Date	N
Epipaleolithic	Hunter-gatherers	Ohalo[1]	35-40	19,000 BP	1
		Ein Mallaha[2]	31.2[3]	14,300-11,500 cal PB	2
Pre-Pottery Neolithic	Hunting, incipient herding, fishing, and farming	Atlit Yam[4]	32.1[3]	9,250-8,000 cal BP	5
Chalcolithic	Farmers and Herders	Peqi'in Cave[5]	32[6]	6,000-5,300 BP	17

Table S2| The sample recent humans[#] included in the various analyses.

**Human evolution (Fig. 1a)**	**Group studied**	**N**			
		**All**	**Males**	**Females**	
	**18-45 years**	74	35	39	
**Human populations characterized by different subsistence strategies (Fig. 1b)[$]**		70	32	38	
**Recent humans with and without fracture according to their bone health (based on the DEXA scores) (Fig. 2)**			**Normal**	**Osteopenia**	**Osteoporosis**
	**Fracture**	**N**	**2**	**16**	**11**
		**Mean Age**	76.5	75.3	73.3
		**±SD**	±2.12	±7.49	±5.16
	**No Fracture**	**N**	**5**	**15**	**10**
		**Mean Age**	59.6	68.8	70.6
		**±SD**	±8.26	±9.11	±7.80
**Human evolution and hip fracture (Fig. 3)**	**Non-osteoporotic Fracture**	**20-40**	**50-60**		
	**No**	54	47		
	**Yes**	NA	3		

[#] Justifications for subsampling the recent (living) population: (1) Differences in life expectancy between the ancient and recent groups - the life expectancy in prehistory (~30 years; Supplementary Table S1) was much lower than that of the Israeli population from which our recent sample derived. Although age had little effect on the PF shape (Table 1), we preferred to examine, as much as possible, groups with similar demographic characteristics. The age of 45 was decided because it is still considered an age with a low prevalence of diseases such as osteoporosis and osteoarthritis[7,8]. (2) Unequal sample size – the *Pan troglodytes*, fossil, ancient, and non-osteoporotic ICHF samples were much smaller than the other recent samples (the control, osteoporotic, and osteopenic groups). Therefore, to reduce bias due to unequal sample sizes, we compared groups with a common age cohort base.

[$]Outliers were excluded following Cardini et al.[9].

Table S3: Pairwise comparisons between groups (*p* and *p* FDR-adj. are presented).

Group	*Pan troglodytes*		Early hominin		Early *Homo*		Neanderthals		Recent HS	
	p	*p (adj.)*	*p*	*p (adj.)*	*p*	*p (adj.)*	*p*	*p (adj.)*	*p*	*p (adj.)*
Pan troglodytes	1	1	0.552	1	0.396	1	0.69	1	0.002	0.025
Early hominin	0.552	1	1	1	0.533	1	0.942	1	0.794	1
Early *Homo*	0.396	1	0.533	1	1	1	0.712	1	0.607	1
Neanderthal	0.69	1	0.942	1	0.712	1	1	1	0.835	1
Recent HS	0.002	0.025	0.794	1	0.607	1	0.835	1	1	1

Table S4: Classification of prehistoric and recent humans into groups using linear discriminant analysis (the Jack-knife method) on the nine first Principal Components (explaining 70.3% of the shape variance obtained from a Principal Component Analysis of the shape space.

**Group**	**Prehistoric**	**Recent humans with ICHF**	**Recent humans**
Prehistoric	87.5%	0.0%	12.5%
Recent humans	11.3%	15.5%	73.2%

References

2. Supplementary Figures

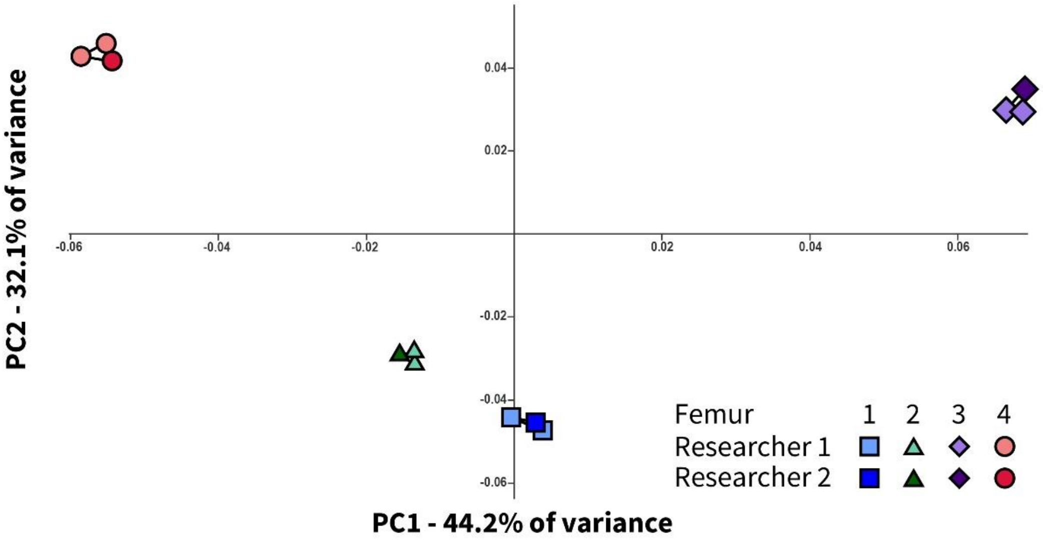

Figure S1| Principal component analysis of the shape variance of the proximal femur (PF) of four individuals. For the intra-observer variation, a single researcher carried out two landmark sessions with a one-week interval between them. For the inter-observer variation, an additional researcher conducted a landmark session on the same proximal femora. Different landmark sessions of the same individuals were grouped; they were significantly closer to each other than to other specimens. No significant difference in landmark placement was found between researchers.

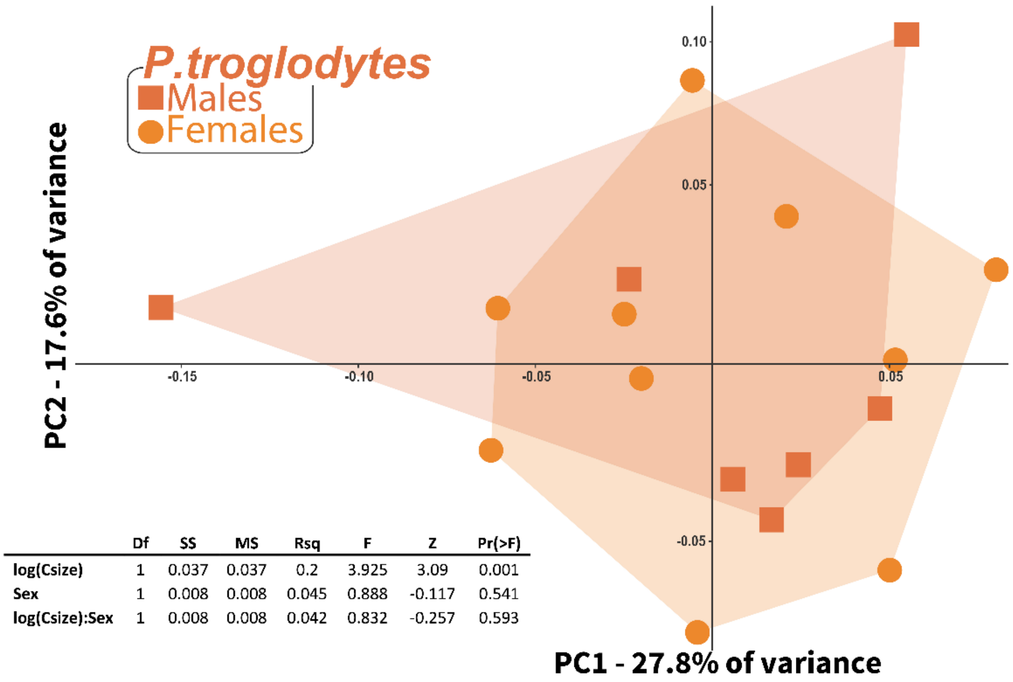

	Df	SS	MS	Rsq	F	Z	Pr(>F)
log(Csize)	1	0.037	0.037	0.2	3.925	3.09	0.001
Sex	1	0.008	0.008	0.045	0.888	-0.117	0.541
log(Csize):Sex	1	0.008	0.008	0.042	0.832	-0.257	0.593

Figure S2| Principal Component Analysis (PCA) plot in shape space for the proximal femur of *Pan troglodytes* by sex (Dots – females; Square – males) and the results of the Procrustes ANOVA. No significant difference was found between the groups via pairwise comparison. According to the Procrustes Anova, sex explained only 4.5% of the shape variance.

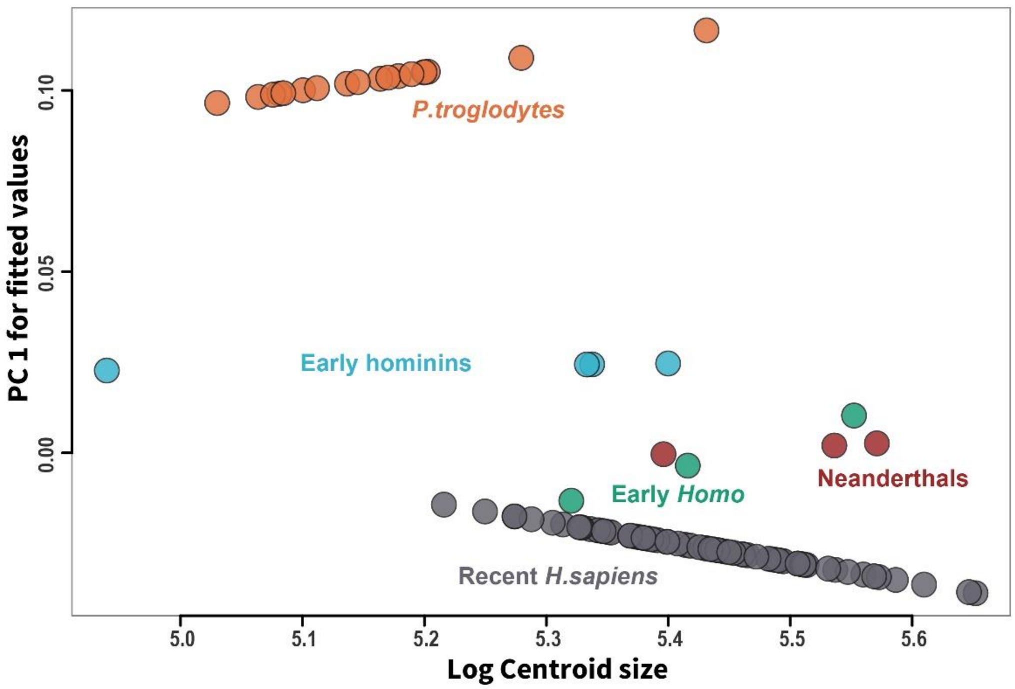

Figure S3| Plot of the predicted shapes against the logarithm of the centroid size among different hominid groups. *Pan troglodytes*: orange; early hominins: blue; early *Homo*: green; Neanderthals: red; and recent humans: gray. Note that the trajectories of humans and *Pan troglodytes* do not share a common allometric trajectory.

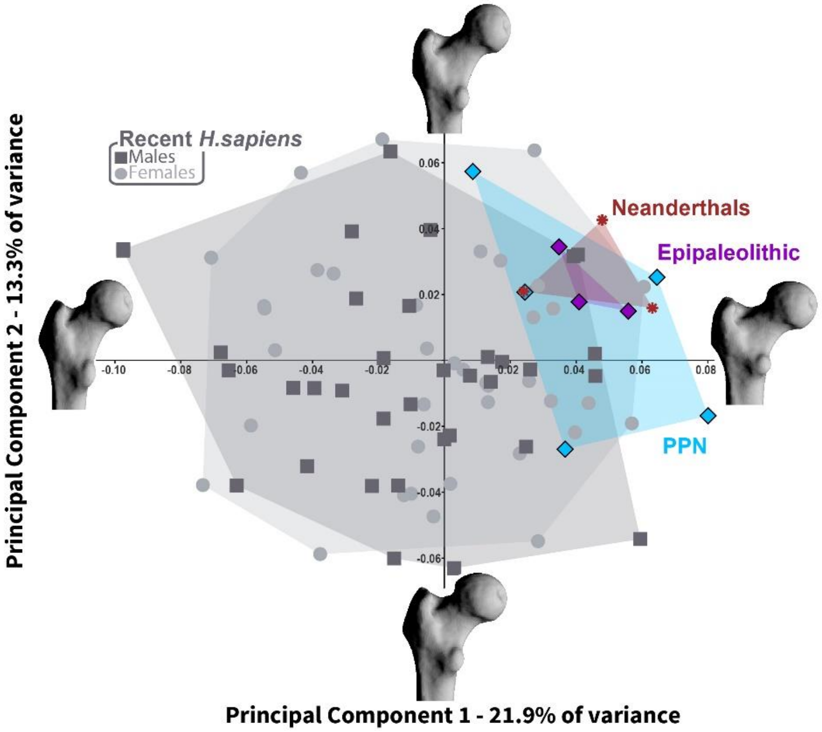

Figure S4| Principal Component Analysis (PCA) plot in shape space for the proximal femur of Neanderthals, Epi-Paleolithic, early farmers, and recent humans (aged 18-45 years).

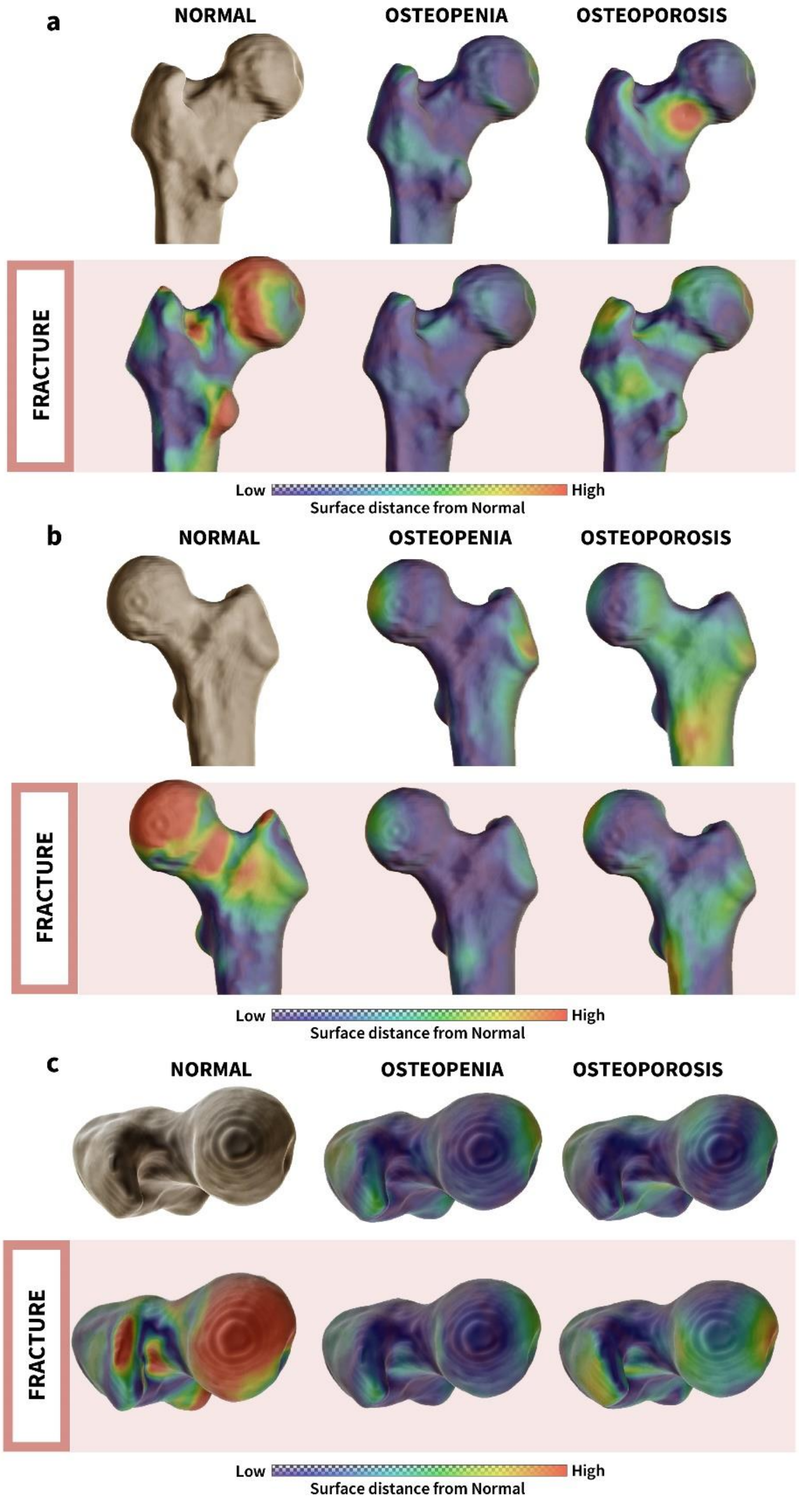

Figure S5| Surface distances represented by colors (blue for the lowest differences and red for the largest differences) of the mean shape of recent human groups divided according to their bone health, defined by the DEXA scores (normal, osteopenia, and osteoporosis) and the presence of fractures (yes/no) from the mean shape of the 'normal' proximal femur (non-osteoporotic, not fractured) from the posterior (**a**), anterior (**b**), and superior (**c**) views.

References

1. Hershkovitz, I. *et al.* Ohalo II H2: A 19,000-year-old skeleton from a water-logged site at the Sea of Galilee, Israel. *Am. J. Phys. Anthropol.* **96**, 215–234 (1995).

2. Valla, F. R., Khalaily, H., Samuelian, N. & Bocquentin, F. From foraging to farming. The contribution of the Mallaha (Eynan) excavations, 1996–2001. *Bull. du Cent. Rech. français à Jérusalem* 71–90 (2002).

3. Hershkovitz, I. & Gopher, A. Demographic, biological and cultural aspects of the neolithic revolution: A view from the southern Levant. in *The Neolithic demographic transition and its consequences* (eds. Bocquet-Apple, J. P. & Bar-Yosef, O.) 441–479 (Springer, 2008).

4. Galili, E., Horwitz, L. K., Eshed, V., Rosen, B. & Hershkovitz, I. Submerged prehistoric settlements off the Mediterranean Coast of Israel. *Skyllis* **13**, 181–204 (2013).

5. Shalem, D., Gal, Z. & Smithline, H. *Peqi'in: A Late Chalcolithic Burial Site Upper Galilee, Israel*. (Kinneret Academic College Institute for Galilean Archaeology, 2013).

6. Nagar, Y. The physical anthropology of the Peqi'in populaiton. in *Peqi'in. A late Chalcolithic Burial Site, Upper Galilee, Israel* (eds. Shalem, D., Gal, Z. & Smithline, H.) 379–393 (Kinneret Academic College Institute for Galilean Archaeology, 2013).

7. Hernandez, C. J., Beaupré, G. S. & Carter, D. R. A theoretical analysis of the relative influences of peak BMD, age-related bone loss and menopause on the development of osteoporosis. *Osteoporos. Int.* **14**, 843–847 (2003).

8. Cross, M. *et al.* The global burden of hip and knee osteoarthritis: estimates from the global burden of disease 2010 study. *Ann. Rheum. Dis.* **73**, 1323–1330 (2014).

9. Cardini, A., Seetah, K. & Barker, G. How many specimens do I need? Sampling error in geometric morphometrics: testing the sensitivity of means and variances in simple randomized selection experiments. *Zoomorphology* **134**, 149–163 (2015).
