# Supplementary material for: Evolutionary roots of the risk of hip fracture in humans
Source: Commun Biol. 2023 Mar 17;6:283. doi: 10.1038/s42003-023-04633-4 (PMC10023703; doi:10.1038/s42003-023-04633-4)
Supplement: Supplementary file 3 — Reporting Summary [file 42003_2023_4633_MOESM3_ESM.pdf]

Corresponding author(s): May Hila

Last updated by author(s): 22/01/2023

Reporting Summary

Nature Portfolio wishes to improve the reproducibility of the work that we publish. This form provides structure for consistency and transparency in reporting. For further information on Nature Portfolio policies, see our Editorial Policies and the Editorial Policy Checklist.

Statistics

For all statistical analyses, confirm that the following items are present in the figure legend, table legend, main text, or Methods section.

n/a	Confirmed	
☐	☒	The exact sample size (*n*) for each experimental group/condition, given as a discrete number and unit of measurement
☐	☒	A statement on whether measurements were taken from distinct samples or whether the same sample was measured repeatedly
☐	☒	The statistical test(s) used AND whether they are one- or two-sided<br>*Only common tests should be described solely by name; describe more complex techniques in the Methods section.*
☐	☒	A description of all covariates tested
☐	☒	A description of any assumptions or corrections, such as tests of normality and adjustment for multiple comparisons
☐	☒	A full description of the statistical parameters including central tendency (e.g. means) or other basic estimates (e.g. regression coefficient) AND variation (e.g. standard deviation) or associated estimates of uncertainty (e.g. confidence intervals)
☐	☒	For null hypothesis testing, the test statistic (e.g. *F*, *t*, *r*) with confidence intervals, effect sizes, degrees of freedom and *P* value noted<br>*Give P values as exact values whenever suitable.*
☒	☐	For Bayesian analysis, information on the choice of priors and Markov chain Monte Carlo settings
☐	☒	For hierarchical and complex designs, identification of the appropriate level for tests and full reporting of outcomes
☒	☐	Estimates of effect sizes (e.g. Cohen's *d*, Pearson's *r*), indicating how they were calculated

Our web collection on statistics for biologists contains articles on many of the points above.

Software and code

Policy information about availability of computer code

Data collection	The three-dimensional shape of the proximal femur was represented using 12 landmarks and 31 curve semi-landmarks (which were placed on four curves). The landmarks, curves, and curve semi-landmarks were placed either manually on the proximal femoral surface mesh using EVAN Toolbox software (v.1.71; www.evan-society.org) or semi-automatically with a dedicated software developed in MATLAB R2014 and is available via https://github.com/nirsh1/Bone-Analysis.git. Semi-landmark sliding along the curves was carried out to minimize thin-plate spline (TPS) bending energy between the target and template using EVAN Toolbox.
Data analysis	Statistical analysis was carried out using PAST and R using the the geomorph and heatmap.2 libraries.

For manuscripts utilizing custom algorithms or software that are central to the research but not yet described in published literature, software must be made available to editors and reviewers. We strongly encourage code deposition in a community repository (e.g. GitHub). See the Nature Portfolio guidelines for submitting code & software for further information.

Data

Policy information about availability of data

All manuscripts must include a data availability statement. This statement should provide the following information, where applicable:

- Accession codes, unique identifiers, or web links for publicly available datasets
- A description of any restrictions on data availability
- For clinical datasets or third party data, please ensure that the statement adheres to our policy

The datasets analyzed during the current study are available in https://figshare.com/s/be2e703abee51789216f. We have no authority to share CT scans of original fossils with a third party. Access to scans of the ancient Homo sapiens sample should be requested from HM. Any other relevant data are available upon reasonable request.

Human research participants

Policy information about studies involving human research participants and Sex and Gender in Research.

Reporting on sex and gender	Differences in proximal femoral shape were examined between the sexes. Since no significant differences were found between males and females, further analyses were carried out for a combined dataset. The sex of the individuals was provided by the medical center. The Helsinki ethic committee of the medical center (approval #432985) confirmed gathering this type of information. Analyses included 188 females and 119 males. Sex of the recent humans included in this study is available in the datasets accompanying this manuscript.
Population characteristics	The 307 recent humans that were included in the study were between 18 and 85 years old. Out of this sample, 82 individuals had an intra-capsular hip fracture. DEXA scores were available for 59 individuals (29 with a hip fracture and 30 without) and were used for bone health categorization (healthy, osteopenic, and osteoporotic).
Recruitment	Patients who underwent abdomen, pelvis, or lower limb CT scans prior to the study (between 2010 and 2017) at the Carmel Medical Center, Haifa, Israel (Brilliance 64, Philips Medical System, Cleveland, Ohio), for medical purposes unrelated to the study. Inclusion criteria was that the entire proximal femora was scanned and that patients are over 18 years. Individuals that showed unfused femoral head, bone pathologies (e.g., bone neoplasms and hip joint arthroplasty not related to hip fracture) or had suffered from locomotor disability acquired prior to the fracture, or had undergone amputations to the lower limb were excluded from the study.
Ethics oversight	The ethics committee of Carmel Medical Center (approval #432985) as well as by the ethics committee of TAU (approval #0000252-1).

Note that full information on the approval of the study protocol must also be provided in the manuscript.

Field-specific reporting

Please select the one below that is the best fit for your research. If you are not sure, read the appropriate sections before making your selection.

☐ Life sciences ☐ Behavioural & social sciences ☒ Ecological, evolutionary & environmental sciences

For a reference copy of the document with all sections, see nature.com/documents/nr-reporting-summary-flat.pdf

Ecological, evolutionary & environmental sciences study design

All studies must disclose on these points even when the disclosure is negative.

Study description	This study aimed to reveal the morphological changes in the proximal femur that increase the risk of intracapsular hip fractures in present-day populations. Using the landmark-based Geometric Morphometric method, we followed shape changes in the proximal femur over time and examined their relationships with intracapsular hip fracture. Our sample included CT scans of Pan troglodytes, early hominins, early Homo, Neanderthals, as well as archaic and recent Homo sapiens. We tested the effect of sex, age, and group affiliation on the proximal femoral shape variance using Procrustes ANOVA, examined shape variation using Principle Component Analysis, and calculated Procrustes distances between each individual to the mean shape of each group. We presented the average distance of each group from the mean shape of each group in a heatmap and dendrogram.
Research sample	This study included virtual reconstructions of the femora of Pan troglodytes (N=18), African Lower Paleolithic hominins (N=7), European Neanderthals (N=3), and Homo sapiens (25 ancient and 307 recent). CT scans of original fossils included an Australopithecus afarensis (A.L. 288-1), two Paranthropus robustus (SK82 and SK97), an unspecified taxon related to either Australopithecus afarensis or Paranthropus robustus (KNM-ER 1503), three early Homo (KNM-ER 1472, KNM-ER 1481, and KNM-WT15000), and three Neanderthals (Krapina 213, Krapina 214, and Neanderthal 1).<br>The archaic Homo sapiens included three groups: Prehistoric Epi-Paleolithic hunter-gatherers (ca. 19,000-11,000 cal BP; N=3), Prehistoric Final Pre-Pottery Neolithic early farmers (9,250–8,000 cal BP PPNC; N=5), and Protohistoric Chalcolithic farmers and herders (6,000-5,300 BP; N=17).

	The recent Homo sapiens sample included proximal femora of 307 recent humans (188 females and 119 males aged 18-85 years old) obtained from medical CT scans. Of these scans, 82 individuals, 61 females (74.0±6.56 years old), and 21 males (71.0±8.37 years old), manifested an intracapsular hip fracture.<br>To evaluate changes in proximal femoral shape throughout human evolution changes in shape variance were examined between Pan troglodytes, early hominins, Neanderthals, and archaic and recent humans. The effect of demographic characteristics and bone health on proximal femoral shape variance was examined by analyzing the data obtained from the recent human sample. To test how changes that occurred during human evolution are related to the risk of hip fracture in recent humans, differences between all groups were examined.
Sampling strategy	Pan troglodytes: All proximal femora available from the Digital Morphology Museum, KUPRI in 2015 were included in the study.<br>Fossils: All fossils with a complete proximal femur that were available at the digital collection of the Max Planck Institute for Evolutionary Anthropology, Leipzig, Germany were included in the study.<br>Archaic Homo sapiens: Only complete proximal femora of prehistoric and protohistoric individuals housed at the Anthropological Collection, Dan David Center for Human Evolution and Bio-history Research, Sackler Faculty of Medicine, Tel Aviv University, were included in the study.<br>Recent sample: CT scans of individuals that followed our inclusion and exclusion criteria (see above).
Data collection	The surface of the proximal femur was generated either by segmentation from the CT stacks using Amira (v. 6.3; www.fei.com) or by surface scanning (Solutionix-Rexcan SC+2; Solutionix, Seoul, South Korea). For the latter, the reconstruction and alignment of the 3D surface of the bone were carried out using EZScan 7 software (Solutionix, Seoul, South Korea). Segmentation and scanning was carried out by Hadas Avni and Victoria Roul.<br>Landmarks (n=12), curves (n=4), and curve semi-landmarks (n=31) were placed manually by Hadas Avni on the proximal femoral surface mesh using EVAN Toolbox software (v.1.71; www.evan-society.org) or semi-automatically with a dedicated software developed in MATLAB R2014 (code available via https://github.com/nirsh1/Bone-Analysis.git).
Timing and spatial scale	Data was collected between 2015 and 2018.
Data exclusions	Data exclusion: Incomplete proximal femora were excluded from the study as well as those demonstrating pathological signs (e.g., severe osteoarthritis in the femoral head). For recent humans, CT scans of individuals that showed bone pathologies (e.g., bone neoplasms and hip joint arthroplasty not related to hip fracture) or had suffered from locomotor disability acquired prior to the fracture, or had undergone amputations to the lower limb were excluded from the study.
Reproducibility	Reliability analyses were carried out by examining Intra- and inter-observer variations in the shape of femoral landmark configurations were assessed using four randomly selected femora. To assess the intra-observer variation, one researcher placed the landmarks and curve semi-landmarks three times on each of the femora with a week-long interval between landmarking sessions. To assess the inter-observer variation, the set of landmarks was placed by an additional independent researcher.
Randomization	Fossils were affiliated with a certain species based on previous publications. Ancient Homo sapiens were affiliated to groups based on dating and subsistence strategy, which were acquired from previous publications. Recent Homo sapiens were divided into groups based on hip fracture manifestation, age, and bone health (normal, osteopenic, and osteoporotic) based on DEXA scores and medical files.
Blinding	As this is a retrospective study blinding was not applicable. We examined proximal femoral shape variation among recent humans who fractured their hip and various other groups.

Did the study involve field work? ☐ Yes ☒ No

Reporting for specific materials, systems and methods

We require information from authors about some types of materials, experimental systems and methods used in many studies. Here, indicate whether each material, system or method listed is relevant to your study. If you are not sure if a list item applies to your research, read the appropriate section before selecting a response.

Materials & experimental systems

n/a	Involved in the study
☒	☐ Antibodies
☒	☐ Eukaryotic cell lines
☒	☐ Palaeontology and archaeology
☐	☒ Animals and other organisms
☒	☐ Clinical data
☒	☐ Dual use research of concern

Methods

n/a	Involved in the study
☒	☐ ChIP-seq
☒	☐ Flow cytometry
☒	☐ MRI-based neuroimaging

Animals and other research organisms

Policy information about studies involving animals; ARRIVE guidelines recommended for reporting animal research, and Sex and Gender in Research

Laboratory animals	The study did not involve laboratory animals
Wild animals	The study did not involve wild animals in nature. It involve CT scans of Pan troglodytes freely available at KUPRI - a Digital Morphology Museum.
Reporting on sex	Sex of the Pan troglodytes was obtained from the Digital Morphology Museum, KUPRI. Shape variance of the proximal femur of Pan troglodytes' males and females was examined in our study.
Field-collected samples	The study did not involve samples collected from the field.
Ethics oversight	The study included CT scans of Pan troglodytes that are freely available via the Digital Morphology Museum, KUPRI (http://dmm.pri.kyoto-u.ac.jp/dmm/WebGallery/index.html). We did not involve recruitment of new animals as well as no direct manipulation of the animal. Therefore, no ethical approval or guidance was necessary.

Note that full information on the approval of the study protocol must also be provided in the manuscript.
